# Multi-omics integration identifies APOE as a metabolic regulator of macrophage-fibroblast crosstalk in idiopathic pulmonary fibrosis

**DOI:** 10.3389/fimmu.2026.1904638

**Published:** 2026-08-18

**Authors:** Bing Bai, Le Chang, Zhi Cheng, Yingjie Wang, Chenfeng Hua, Yonghai Feng, Wenfei Zhao

**Affiliations:** 1 Department of General Practice, Fuhua Street Community Health Service Center, The Fifth Affiliated Hospital of Zhengzhou University, Zhengzhou, Henan, China; 2 Henan Key Laboratory for Helicobacter pylori and Digestive Tract Microecology, The Fifth Affiliated Hospital of Zhengzhou University, Institute of Rehabilitation Medicine, Henan Academy of Innovations in Medical Science, Zhengzhou, Henan, China; 3 Hepatobiliary Pancreatic Surgery, The Fifth Affiliated Hospital of Zhengzhou University, Zhengzhou, Henan, China; 4 Key Laboratory of Tobacco Chemistry, Zhengzhou Tobacco Research Institute of China National Tobacco Corporation (CNTC), Zhengzhou, Henan, China; 5 Department of Respiratory and Critical Care Medicine, The Fifth Affiliated Hospital of Zhengzhou University, Zhengzhou, Henan, China

**Keywords:** APOE, idiopathic pulmonary fibrosis, macrophage polarization, Mendelian randomization, single-cell RNA sequencing

## Abstract

**Background:**

Aberrant tissue repair and relentless fibroblast activation are hallmark features of idiopathic pulmonary fibrosis (IPF). Although IPF and Alzheimer’s disease (AD) share underlying aging-related pathologies, including immune and metabolic dysregulation, the putative genetic mechanisms linking AD susceptibility to pathogenic macrophage remodeling in the fibrotic niche are not fully established.

**Methods:**

We performed a two-sample Mendelian randomization (MR) analysis to assess the genetic association and potential causal relationship between AD and IPF. Shared hub genes were identified via protein interaction networks. To characterize macrophage heterogeneity and intercellular crosstalk within the IPF microenvironment, we interrogated scRNA-seq data (GSE122960) utilizing Monocle 3 and CellChat algorithms. The functional essentiality of APOE was evaluated bridging computational virtual knockout (scTenifoldKnk) with laboratory *in vitro* assays. Specifically, downstream transcriptomic shifts and fibroblast activation capacities were validated using APOE-silenced THP-1 macrophages and a Transwell co-culture model with MRC-5 cells.

**Results:**

MR estimates indicated that genetic liability to AD is associated with a lower risk of developing IPF. Integrated profiling identified the lipid-metabolism gene APOE as a central hub, specifically enriched in lung macrophages. Pseudotime modeling captured a pathogenic bifurcation in IPF, where macrophages evolve toward a terminal state marked by profound oxidative phosphorylation defects and massive SPP1 secretion. These SPP1+ macrophages primarily activate fibroblasts via CD44 and integrin signaling axes. Furthermore, both virtual simulations and *in vitro* THP-1 experiments demonstrated that loss of APOE function triggers the hyperactivation of complement (C1QA) and antigen-presentation (HLA-DR) pathways. Co-culture assays ultimately confirmed that APOE ablation in macrophages strongly exacerbates myofibroblast differentiation (elevated α-SMA and collagen I) in adjacent MRC-5 cells.

**Conclusion:**

APOE functions as a vital metabolic barrier against pro-fibrotic macrophage polarization in the lung. Disruption of this specific lipid metabolic network is strongly associated with SPP1-driven fibroblast activation and local immune imbalance, providing a theoretical framework that strictly warrants future *in vivo* investigation to determine its clinical relevance.

## 1 Introduction

Idiopathic pulmonary fibrosis (IPF) relentlessly destroys lung architecture through runaway extracellular matrix (ECM) deposition and irreversible structural remodeling (1).While current antifibrotic therapies, including pirfenidone and nintedanib, provide some clinical benefit by slowing functional decline, they fail to halt or reverse the underlying fibrotic cascade (2). At the core of this refractory pathology is a vicious cycle of recurrent alveolar injury, oxidative stress, and maladaptive tissue repair. Within this disrupted local niche, the immune microenvironment-and specifically the phenotypic reprogramming of lung macrophages-emerges as a primary orchestrator of persistent myofibroblast activation and matrix deposition (3).

Intriguingly, the clinical trajectory of IPF closely parallels certain neurodegenerative conditions, positioning it as a profound aging-associated disorder. Although immune dysregulation and metabolic disturbances are common features of several age-related neurodegenerative diseases, including Parkinson**’**s disease and amyotrophic lateral sclerosis, Alzheimer**’**s disease (AD) was specifically prioritized in the present study because of its close association with dysregulated lipid metabolism. Overlapping epidemiological and transcriptomic evidence suggests that IPF and AD share biological vulnerabilities related to metabolic dysfunction, mitochondrial impairment, and cellular senescence (4, 5). Notably, apolipoprotein E (APOE), the strongest genetic risk factor for late-onset AD and a central regulator of lipid transport and cholesterol homeostasis, has also emerged as an important regulator of macrophage lipid handling, immunometabolism, oxidative stress, and tissue remodeling, providing a biologically plausible mechanistic link between AD and IPF. The growing recognition of cognitive impairment among patients with IPF further supports the existence of systemic pathological crosstalk between pulmonary fibrosis and neurodegeneration (6). Lipid homeostasis appears to be a critical molecular bridge connecting these two distinct entities. Apolipoprotein E (APOE), classically studied as a central driver of AD pathogenesis via cholesterol transport, is gaining attention as a potent modulator of macrophage immunometabolism and oxidative stress in the pulmonary niche (7). When local lipid metabolism fails, lipid-laden macrophages lose their reparative plasticity, trapping the lung in a state of chronic inflammation and active fibrogenesis (8). However, whether genetic susceptibility to AD causally influences the risk of IPF, and how APOE-mediated macrophage dysfunction contributes to pulmonary fibrosis, remain largely unknown.

The advent of high-resolution single-cell RNA sequencing (scRNA-seq) has revolutionized our understanding of the fibrotic lung, capturing cellular heterogeneity at an unprecedented scale (9). A major breakthrough from these transcriptomic atlases is the identification of a highly destructive, SPP1-overexpressing macrophage subpopulation (10). Dense accumulations of these SPP1+ macrophages physically localize to fibrotic foci, where they continuously feed paracrine signals to drive fibroblast activation and epithelial–mesenchymal transition (EMT) (11, 12). Despite this precise spatial mapping, the dynamic temporal trajectory forcing steady-state macrophages into this terminal pathogenic phenotype is not fully mapped. More importantly, it remains unknown how the breakdown of APOE-mediated metabolic checkpoints precipitates this catastrophic phenotypic shift in fibrotic environments.

In the present study, we combined genetic epidemiology with single-cell transcriptomic analyses to investigate the relationship between AD-associated genetic traits and IPF. Mendelian randomization (MR) analysis was performed using large-scale genome-wide association study (GWAS) datasets to evaluate the potential causal association between AD and IPF susceptibility. After identifying APOE as a shared hub gene, we further analyzed scRNA-seq data (GSE122960) to characterize macrophage heterogeneity and state transitions in the fibrotic lung. By integrating pseudotime trajectory analysis, CellChat-based intercellular communication analysis, and scTenifoldKnk virtual knockout analysis, we aimed to determine how APOE-mediated lipid metabolic regulation influences macrophage polarization and fibroblast activation during pulmonary fibrosis.

## 2 Materials and methods

### 2.1 Mendelian randomization analysis

Summary statistics from publicly available genome-wide association study (GWAS) datasets were used for two-sample Mendelian randomization (MR) analysis to evaluate the association between Alzheimer**’**s Disease-related traits and Idiopathic Pulmonary Fibrosis risk. To minimize potential bias due to population stratification, all exposure and outcome GWAS datasets were derived exclusively from individuals of European ancestry. Instrumental variables (IVs) were selected at genome-wide significance(*P* < 5×10^8^). Linkage disequilibrium (LD) pruning was performed using the 1000 Genomes European reference panel (r^2^<0.001, window=10 Mb). Palindromic and incompatible SNPs were removed during data harmonization. To reduce potential pleiotropic bias, SNPs associated with known confounding factors (e.g., smoking-related traits) or the outcome (IPF) at *P* < 1×10⁻^5^ were identified using the PhenoScanner V2 database and excluded. Directional horizontal pleiotropy was assessed using the MR-Egger intercept test. Instrument strength was evaluated using the F-statistic, and SNPs with an F-statistic > 10 were retained. Causal estimates were primarily calculated using the inverse variance weighted (IVW) method, with MR-Egger regression and the weighted median method performed as complementary sensitivity analyses.

**Figure 1:**
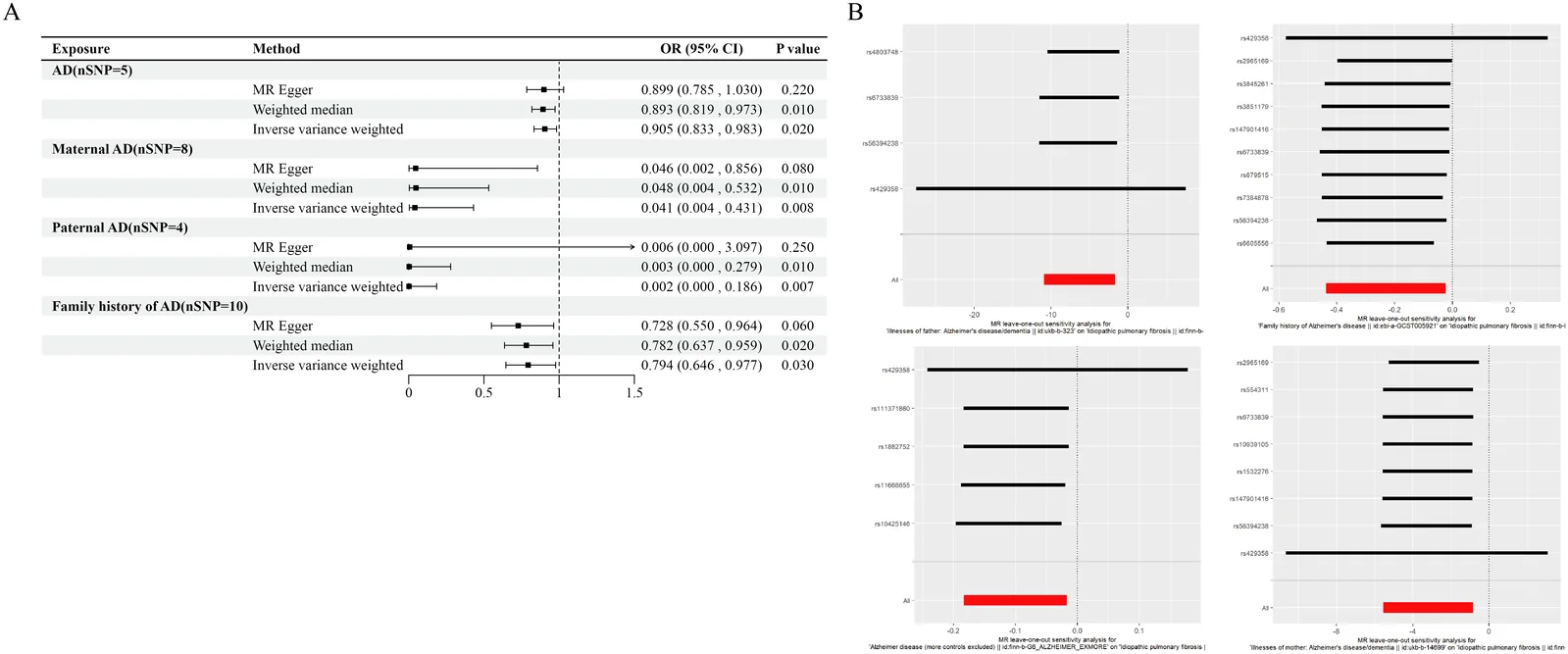
Causal association between Alzheimer’s disease AD and IPF. **(A)** Forest plot showing Mendelian randomization (MR) estimates of AD and AD-related traits on IPF susceptibility. The primary IVW analysis indicates a significantly reduced risk of IPF (OR < 1, *P* < 0.05). **(B)** Leave-one-out sensitivity analyses confirming the robustness of the causal associations, demonstrating that the results are not driven by any single pleiotropic outlier SNP.

Leave-one-out analysis was performed to assess the stability of the results. Detailed characteristics of the exposure and outcome GWAS datasets, including GWAS accession numbers, sample size, ancestry, case-control definitions, and data sources, are summarized in **Table 1**. The final numbers of instrumental SNPs included for each exposure are presented in **Figure 1A** and **Supplementary Table 2**.

**Table 1:** Characteristics of exposures and outcome.

Variable	GWAS ID	Cases	Controls	Sample size	Population
Exposure					
AD	finn-b-G6_ALZHEIMER_EXMORE	3,899	111,471	115,370	European
Maternal AD	ukb-b-14699	36,548	387,190	423,738	European
Paternal AD	ukb-b-323	19,255	380,538	399,793	European
Family history of AD	ebi-a-GCST005921	0	314,278	314,278	European
Outcome					
IPF	finn-b-IPF	1,028	196,986	198,014	European

### 2.2 Protein-protein interaction network and enrichment analysis

Genes associated with the selected IVs were identified using the Functional Mapping and Annotation of Genome-Wide Association Studies (FUMA) platform. These genes were imported into the STRING database to construct a protein-protein interaction (PPI) network and visualized in Cytoscape. Hub genes were identified using the cytoHubba plugin according to topological characteristics across different ranking algorithms.

Gene Ontology (GO) and Kyoto Encyclopedia of Genes and Genomes (KEGG) enrichment analyses were conducted using the clusterProfiler R package. Adjusted *P* < 0.05 was considered statistically significant.

### 2.3 scRNA-seq data processing

To delineate the transcriptomic landscape of the fibrotic lung, we retrieved the GSE122960 scRNA-seq cohort from the Gene Expression Omnibus (GEO). This collection comprises pulmonary biopsies from both IPF patients and healthy controls. We executed core preprocessing and downstream computational workflows relying on the Seurat R suite. Rigorous quality control filters were applied to discard low-viability events; specifically, we filtered out droplets exhibiting aberrant unique molecular identifier (UMI) metrics, insufficient gene complexity, or excessive mitochondrial transcripts. Following global normalization and variance scaling, we extracted highly variable features to guide principal component analysis (PCA). To visualize the complex multidimensional data and define cluster topologies, we projected the cellular subsets onto two-dimensional spaces utilizing UMAP and t-SNE algorithms. Finally, distinct cellular populations were assigned their biological identities by mapping canonical lineage-specific marker genes.

### 2.4 Pseudotime trajectory analysis

To map the continuous developmental transitions within the monocyte-macrophage lineage, we applied the Monocle 3 algorithmic framework. We specifically isolated monocyte and macrophage subsets from the integrated scRNA-seq dataset to construct differentiation trajectories. By anchoring monocytes as the developmental root, we chronologically ordered the remaining cell populations based on their transcriptomic proximity. To track the dynamic molecular shifts accompanying macrophage maturation, we projected the expression signatures of canonical immune, lipid metabolic, and pro-fibrotic regulators onto spatial feature plots and pseudotime heatmaps.

### 2.5 Cell-cell communication analysis

We employed the CellChat R package to infer complex intercellular crosstalk across the fibrotic niche. Taking normalized transcript matrices and refined cell annotations as input, we modeled the probability of ligand-receptor communication events, directing our primary focus toward macrophage-fibroblast interactions. We specifically interrogated the signaling intensity of established fibrogenic cascades-such as the SPP1 and TGF-β pathways-and mapped these directional communication networks using custom hierarchical heatmaps, circle plots, and paired bubble charts.

### 2.6 Virtual gene knockout analysis

To computationally predict the biological consequences of APOE functional loss, we leveraged the scTenifoldKnk machine-learning pipeline. We initially built robust single-cell gene regulatory networks (scGRNs) exclusively from the macrophage subpopulations. Next, we introduced an in silico perturbation to silence the APOE node. By aligning the manifolds of the simulated knockout and wild-type networks, we quantified the systemic regulatory shifts. Significant perturbation targets were extracted relying on stringent fold-change and Z-score thresholds, which we subsequently subjected to GO and KEGG enrichment mapping to uncover the broader transcriptomic impact of APOE depletion.

### 2.7 Cell culture and macrophage differentiation

We sourced the human THP-1 monocytic cell line and MRC-5 lung fibroblasts from Procell Life Science & Technology Co., Ltd. For routine propagation, we maintained THP-1 suspensions in RPMI-1640 medium, while expanding the adherent MRC-5 cultures in DMEM. All base media were enriched with 10% fetal bovine serum (FBS) and a 1% penicillin-streptomycin cocktail. We continuously housed the cell lines under standard physiological conditions within a humidified incubator maintained at 37 °C and CO_2_. THP-1 monocytes were differentiated into macrophage-like cells by treatment with 50 ng/mL phorbol 12-myristate 13-acetate (PMA) for 48 h. Cells were then cultured in fresh complete medium for an additional 24 h before subsequent experiments.

### 2.8 siRNA transfection

APOE knockdown was achieved using APOE-specific siRNA (Sense: 5′-GGCCAAUCACAGGCAGGAAUU-3′; Antisense: 5′-UUCCUGCCUGUGAUUGGCCUU-3′). Scrambled siRNA was used as a negative control. All siRNAs were synthesized by GenePharma (Shanghai, China). Transfection was performed using Lipofectamine RNAiMAX according to the manufacturer’s protocol. Cells were collected 48 h after transfection for qPCR and Western blot analyses.

### 2.9 Macrophage-fibroblast co-culture assay

A Transwell co-culture system (0.4 μm pore size; NEST Biotechnology, Wuxi, China) was established to investigate the impact of APOE-deficient macrophages on fibroblast activation. MRC-5 fibroblasts were plated in the lower compartment, while THP-1-derived macrophages transfected with APOE siRNA or negative control siRNA were placed in the upper inserts. Following 48 h of indirect co-culture, MRC-5 cells were collected to assess the expression levels of α-SMA and collagen I.

### 2.10 RNA extraction, qPCR, and western blotting

Total RNA was extracted using TRIzol reagent (Invitrogen, Thermo Fisher Scientific, Waltham, MA, USA). Complementary DNA (cDNA) was synthesized using a reverse transcription kit (Vazyme Biotech, Nanjing, China). Quantitative real-time PCR (qPCR) was performed using SYBR Green master mix to measure the expression levels of APOE, C1QA, HLA-DR, α-SMA, TNF-α, TGF-β1 and collagen I. Relative gene expression was calculated using the 2^-ΔΔCt^ method with GAPDH as the internal reference. Primer sequences are listed in **Supplementary Table 1**.

For Western blotting, proteins were extracted using RIPA lysis buffer containing protease inhibitors. Equal amounts of protein were separated by SDS-PAGE and transferred onto PVDF membranes. Membranes were incubated overnight at 4 °C with primary antibodies against APOE (Abcam, Cambridge, UK; Cat. No. ab52607, 1:1000), C1QA (Proteintech, Wuhan, China; Cat. No. 14626-1-AP, 1:1000), HLA-DR (Abcam, Cambridge, UK; Cat. No. ab92511, 1:1000), α-SMA (Cell Signaling Technology, Danvers, MA, USA; Cat. No. 19245, 1:1000), Collagen I (Abcam, Cambridge, UK; Cat. No. ab34710, 1:1000), and GAPDH (Proteintech, Wuhan, China; Cat. No. 60004-1-Ig, 1:5000). After incubation with HRP-conjugated secondary antibodies, protein bands were detected using an enhanced chemiluminescence system.

### 2.11 Statistical analysis

Bioinformatics analyses were performed using R software (version 4.3.2). Experimental data are presented as mean ± standard deviation (SD) from at least three independent experiments. Statistical analyses were conducted using GraphPad Prism (version 10.1.2). Comparisons between two groups were performed using Student’s t-test. A two-sided P < 0.05 was considered statistically significant.

## 3 Results

### 3.1 Genetically predicted AD is associated with a reduced risk of IPF

Two-sample Mendelian randomization (MR) analysis was performed to evaluate the association between Alzheimer**’**s Disease-related traits and Idiopathic Pulmonary Fibrosis susceptibility (**Supplementary Table 2**). The inverse variance weighted (IVW) model demonstrated that genetically predicted AD was significantly associated with a lower risk of IPF (OR = 0.905, 95% CI: 0.833-0.983, *P* = 0.020). Similar protective associations were also observed for maternal AD (OR = 0.041, *P* = 0.008), paternal AD (OR = 0.002, *P* = 0.007), and family history of AD (OR = 0.794, *P* = 0.030) (**Figure 1A**).

The robustness of our primary genetic associations was further verified through a stringent dual-strategy pleiotropy assessment and sensitivity modeling. Prior to the MR estimates, manual screening via the PhenoScanner database successfully excluded SNPs associated with known confounding traits. Furthermore, the MR-Egger intercept test provided robust statistical evidence confirming the complete absence of directional horizontal pleiotropy across all four AD-related exposure traits (AD:*P* = 0.92; Maternal AD:*P* = 0.91; Paternal AD:*P* = 0.61; Family history of AD:*P* = 0.39) (**Table 2**). In parallel, Cochran**’**s Q statistics confirmed the absence of significant heterogeneity across the instrumental variables utilized in our MR framework (IVW:*P* = 0.82; MR-Egger:*P* = 0.68). Sequential leave-one-out testing revealed no fundamental shifts in the overall MR estimates upon the removal of any single variant, effectively excluding the possibility of outlier-driven bias (**Figure 1B**). These metrics jointly affirm the reliability of the inferred AD-IPF genetic association.

**Table 2:** Sensitivity analyses of MR.

Exposure	Heterogeneity test						Pleiotropy test		
	IVW			MR-Egger			MR-Egger intercept		
	Q	Q_df	Q_pval	Q	Q_df	Q_pval	Intercept	SE	P
AD	1.51	4	0.82	1.50	3	0.68	0.006	0.05	0.92
Maternal AD	3.00	7	0.88	2.99	6	0.81	-0.003	0.02	0.91
Paternal AD	2.48	3	0.48	2.11	2	0.36	-0.02	0.03	0.61
Family history of AD	11.28	9	0.26	10.22	8	0.25	0.02	0.02	0.39

### 3.2 APOE is identified as a central hub gene linking AD and IPF

Moving from statistical correlation to biological interaction, we mapped the significant MR variants to their corresponding genomic loci via FUMA and reconstructed the downstream PPI landscape using the STRING database (**Supplementary Figure 1A**). Topological scoring of this network-leveraging six independent algorithms within CytoHubba (MCC, Degree, MNC, EPC, Closeness, and EcCentricity)-consistently isolated APOE as the predominant central hub (**Supplementary Figures 1B–G**).

The reproducibility of this genetic overlap was subsequently tested across four distinct AD-related exposure datasets (encompassing primary, maternal, paternal, and family history traits). Within these diverse cohorts, the rs429358 locus consistently retained high significance and mapped directly to APOE, solidifying its status as a shared pathogenic driver. Functional enrichment profiling of the broader hub network pointed heavily toward lipid transport, cholesterol efflux, and chylomicron clearance (**Supplementary Figure 2**). This integrative screening strategy highlighted APOE as a central metabolic candidate. While network centrality alone does not establish functional causality, this prioritization provided a strong rationale for further investigating the cell-type-specific role of APOE within the fibrotic single-cell landscape and in subsequent*in vitro* functional assays.

### 3.3 The fibrotic niche induces a functional APOE metabolic deficit in macrophages

**Figure 2:**
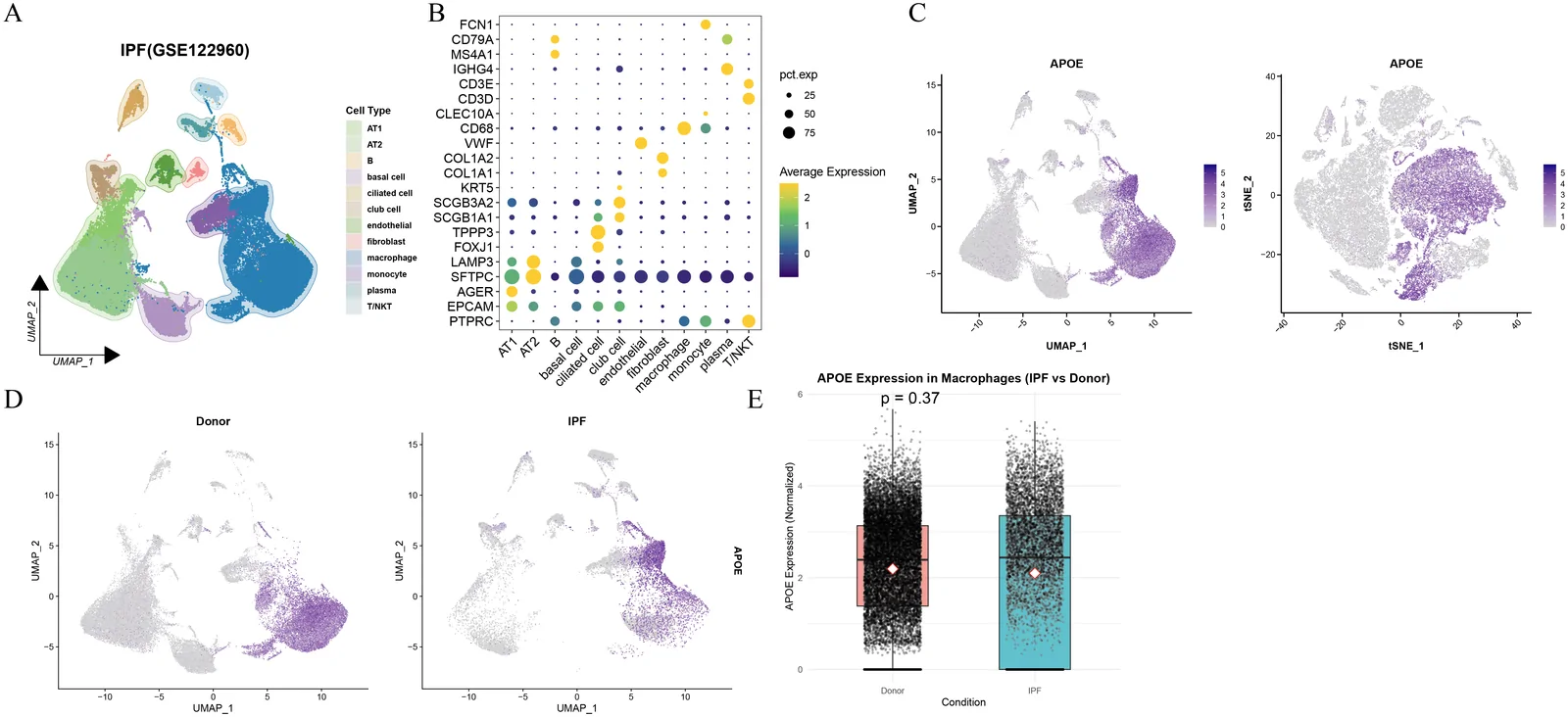
APOE is predominantly expressed in pulmonary macrophages. **(A)** UMAP visualization of diverse cell populations in human lung tissues based on the GSE122960 dataset. **(B)** Dot plot showing the expression of canonical marker genes used for cell-type identification. **(C)** UMAP and t-SNE feature plots revealing that APOE is predominantly and robustly expressed in the macrophage cluster. **(D)** Split UMAP feature plots comparing APOE expression mapping between Donor and IPF conditions. **(E)** Quantitative comparison indicating that the overall APOE expression level within macrophages remains constitutively high and does not significantly differ between normal and IPF lungs (*P* = 0.37).

Given that bulk genetic data lacks spatial and lineage specificity, we leveraged the GSE122960 scRNA-seq cohort to resolve the expression profile of APOE within the IPF microenvironment. Unsupervised classification identified 11 discrete cellular communities, highlighting macrophages as the predominant immune constituent (**Figures 2A, B**). Feature plots confirmed a striking compartmentalization, with APOE transcription overwhelmingly confined to the macrophage pool rather than adjacent structural cells (**Figures 2C, D**).

**Figure 3:**
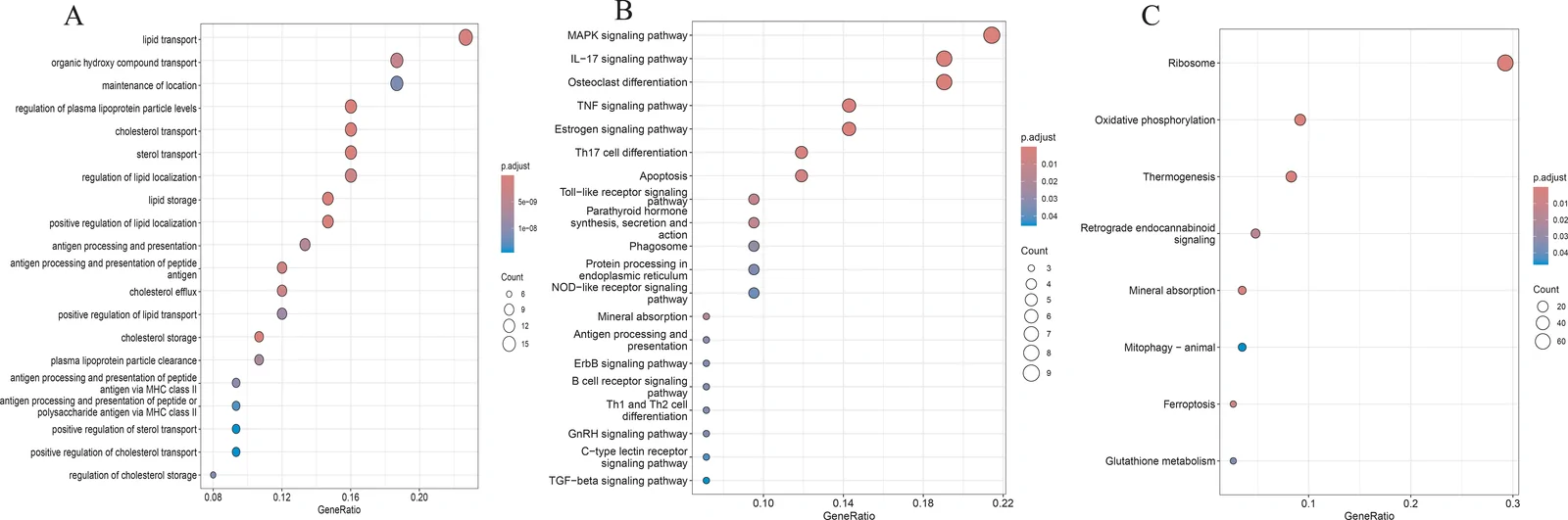
Functional enrichment analysis of differentially expressed genes in fibrotic macrophages. **(A)** Gene Ontology (GO) enrichment analysis of differentially expressed genes. **(B)** Kyoto Encyclopedia of Genes and Genomes (KEGG) pathway enrichment analysis. **(C)** Functional enrichment analysis showing the major biological processes and pathways associated with the differentially expressed genes. In all scatter plots, dot size represents the number of enriched genes, and color indicates the level of statistical significance.

Quantitative comparisons across the clinical cohorts yielded an unexpected finding: the absolute expression levels of APOE in IPF macrophages did not differ significantly from those in healthy controls (*P* = 0.37) (**Figure 2E**). To determine whether APOE expression differences were masked by macrophage heterogeneity, we further analyzed APOE expression across individual monocyte-macrophage states. APOE was broadly expressed across protective, transitional, and pro-fibrotic macrophage populations, with more than 80% of cells showing APOE positivity (**Supplementary Figure 3**). Interestingly, APOE expression was not reduced in the pro-fibrotic subset but showed relatively increased expression intensity, suggesting a potential compensatory transcriptional response under fibrotic stress. However, pathway-level dissection revealed a profound functional divergence. Macrophages maintaining robust APOE signatures exhibited intact lipid homeostasis and transport circuits (**Figure 3A**). Conversely, cells losing these APOE-associated traits suffered from heightened apoptotic stress and robust activation of MAPK/TNF inflammatory cascades (**Figure 3B**). Parallel to this inflammatory shift, essential mitochondrial processes-such as oxidative phosphorylation and mitophagy-were severely compromised in the IPF group (**Figure 3C**). Thus, while APOE mRNA remains physically present, transcriptomic profiling strongly suggests that its associated protective metabolic network is severely impaired by the fibrotic milieu.

### 3.4 Pseudotime-based trajectory analysis reveals macrophage state remodeling toward an SPP1+ pro-fibrotic phenotype

**Figure 4:**
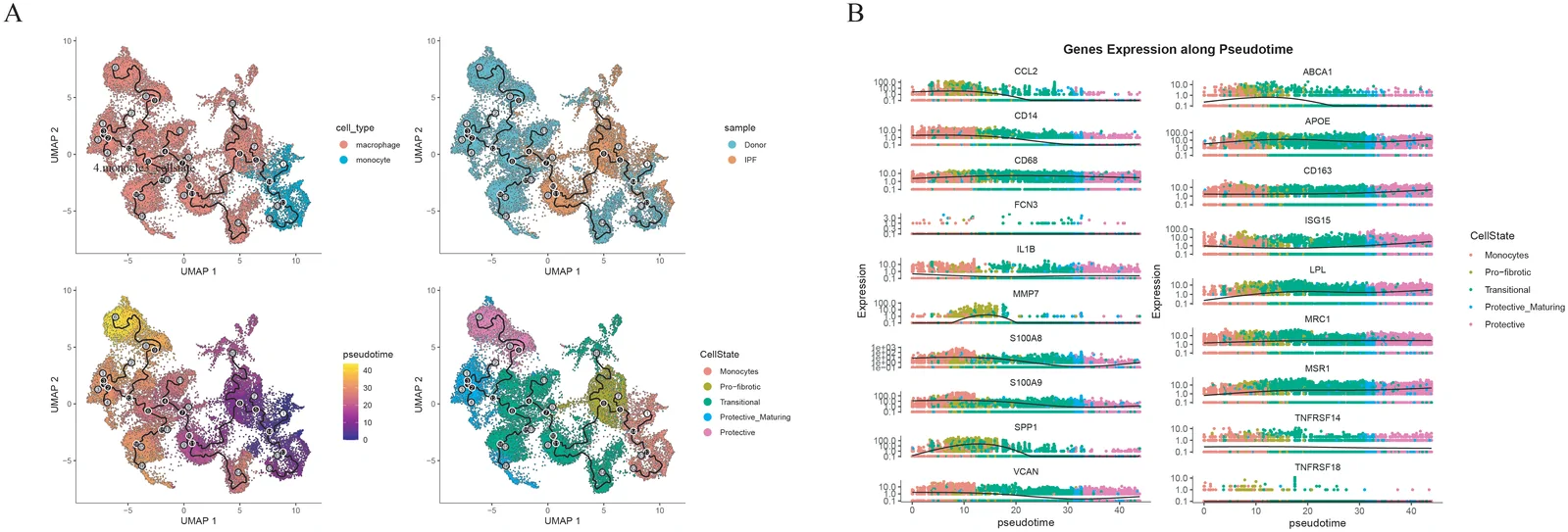
Pseudotime trajectory analysis of macrophage differentiation in IPF. **(A)** Monocle 3 trajectory analysis showing the inferred differentiation trajectory of myeloid cells. Cells are colored according to cell type, disease status, and pseudotime. **(B)** Expression dynamics of representative macrophage functional genes across the inferred pseudotime ordering, reflecting changes in metabolic, inflammatory, and pro-fibrotic macrophage states.

To characterize the dynamic organization of macrophage states associated with fibrosis progression, we performed pseudotime-based trajectory analysis of the monocyte-macrophage compartment using Monocle 3. Monocle 3 inference delineated a potential continuous maturation path based on inferred transcriptomic shifts, tracking the progression from highly CD14-expressing peripheral monocytes into fully differentiated, tissue-resident CD68+/CD163+ macrophages (**Figure 4A**).

Importantly, this continuum featured a prominent pathological bifurcation, culminating in a terminal state heavily enriched with IPF-derived cells (**Figure 4A**). Temporal mapping of gene expression along this disease-specific branch revealed a rapid, late-stage induction of SPP1 and MMP7 (**Figure 4B**)-the classical markers of pro-fibrotic remodeling. Notably, the continuous basal transcription of APOE along this trajectory was insufficient to restrict the transition into the pathogenic SPP1+ phenotype. This spatial-temporal modeling strongly implies that environmental fibrotic stress overwhelms APOE-mediated metabolic buffering, thereby licensing the emergence of matrix-depositing macrophage effectors.

To determine whether the inferred trajectory was influenced by integration of donor and IPF samples, we further performed independent Monocle 3 trajectory analyses in donor and IPF monocyte/macrophage populations. In each condition, monocyte and macrophage populations were independently extracted, dimensional reduction was recalculated, and pseudotime trajectories were reconstructed using monocyte-enriched states as the starting point. These independent analyses demonstrated that a monocyte-macrophage transcriptomic continuum could be reconstructed separately in both donor and IPF samples, indicating that the original trajectory organization was not solely driven by combined analysis of control and IPF cells (**Supplementary Figures 4A–D**). Comparison of IPF samples with donor samples via pseudotime-associated expression analysis revealed dynamic changes in lipid metabolism-related genes (APOE, ABCA1, and LPL) and fibrosis/inflammation markers (SPP1, MMP7, CCL2, and IL1B), suggesting disease-associated remodeling of macrophage functional states (**Supplementary Figures 4E, F**).

Given the substantial heterogeneity of IPF macrophages, we further performed an independent subtype annotation analysis of the macrophage compartment. This analysis identified six canonical macrophage populations, including resident tissue macrophages (RTM), lipid-associated macrophages (LA-M), inflammatory macrophages (Inflam-M), fibrosis-associated macrophages (fibrosis-M), anti-inflammatory macrophages (Anti-Inflam-M), and proliferative macrophages (Prolif-M) (**Supplementary Figure 5**). These results confirmed that the macrophage compartment contains diverse functional populations rather than a single homogeneous trajectory. Therefore, the CellState framework used in the main analysis was intended to capture functional state organization along the monocyte-macrophage continuum, while the subtype annotation provides complementary information regarding established macrophage heterogeneity.

### 3.5 Macrophage-derived SPP1 mediates fibroblast activation through paracrine signaling

To decode the intercellular communication networks fueling fibrogenesis, we applied CellChat modeling to the scRNA-seq dataset. Before performing cell-cell communication inference, we further validated the accuracy of cell-type annotation by examining canonical marker gene expression across the identified cell populations. As shown in **Supplementary Figure 6**, macrophage populations exhibited enriched expression of myeloid/macrophage-associated markers, including LST1, TYROBP, FCER1G, CTSS, CD68, C1QA, C1QC, and MARCO, whereas fibroblast populations showed specific expression of extracellular matrix and fibroblast-associated markers, including COL1A1, COL1A2, COL3A1, DCN, LUM, COL6A1, COL6A2, and PDGFRA. These distinct expression patterns confirmed the reliability of macrophage and fibroblast annotations and provided a robust foundation for subsequent cell–cell communication analysis.

**Figure 5:**
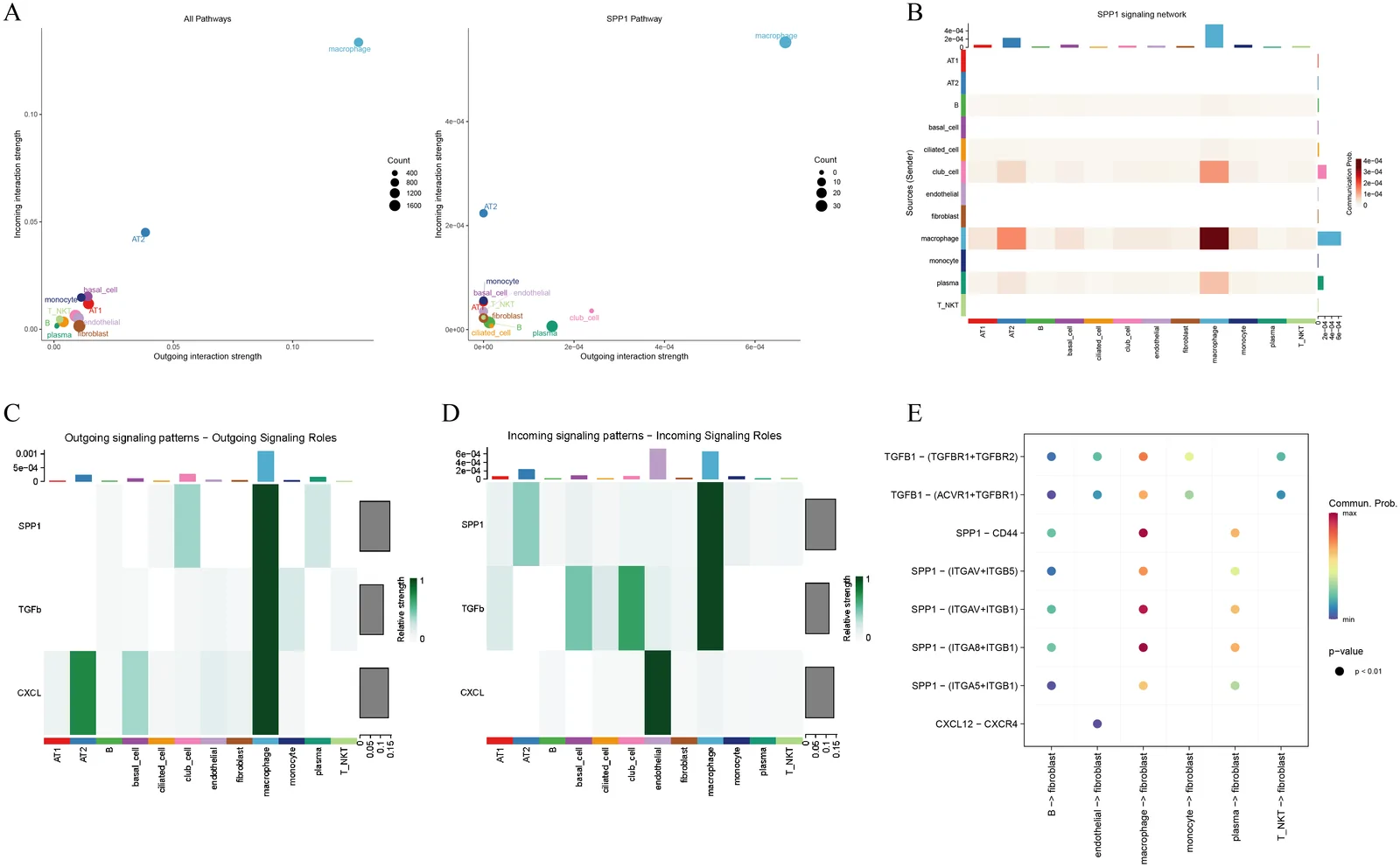
Cell-cell communication analysis between macrophages and fibroblasts in IPF. **(A)** Overall cell–cell communication network showing the number and strength of predicted interactions among major cell populations. **(B–D)** CellChat analysis illustrating predicted outgoing SPP1-mediated signaling from macrophages to neighboring cell populations. **(E)** Predicted ligand–receptor interactions between macrophage-derived SPP1 and its corresponding receptors, including CD44, ITGAV/ITGB5, ITGAV/ITGB1, and ITGA8/ITGB1, in fibroblasts.

CellChat analysis revealed that macrophages represented a major signaling source within the IPF niche, actively communicating with fibroblast populations (**Figure 5A, B**). When dissecting specific profibrotic pathways, the SPP1 signaling axis emerged as the most dominant conduit of macrophage-to-fibroblast crosstalk (**Figures 5C, D**). High-resolution evaluation of ligand-receptor pairing confirmed that this targeted communication relies heavily on the binding of macrophage-secreted SPP1 to fibroblastic CD44, alongside specific integrin complexes such as ITGAV/ITGB1 and ITGAV/ITGB5 (**Figure 5E**). This robust paracrine wiring physically implicates SPP1+ macrophages as key drivers of fibroblast activation.

To further investigate lipid-handling intercellular communication in IPF, we performed a targeted CellChat analysis of prespecified lipid/metabolism-associated signaling families (including ANGPTL, CHEMERIN, PROS, and VISFATIN). This sample-level analysis identified significantly increased fibroblast-to-macrophage PROS signaling in IPF, predominantly mediated by the PROS1-AXL ligand–receptor pair (FDR = 0.039; **Supplementary Figure 7**). Although the current CellChat database does not include the canonical APOE-LRP1 axis, the selective upregulation of PROS1-AXL, a pathway involved in efferocytosis and lipid handling, provides additional support for altered lipid-handling communication between macrophages and fibroblasts in IPF.

### 3.6 APOE deficiency drives the inflammatory and pro-fibrotic reprogramming of macrophages

**Figure 6:**
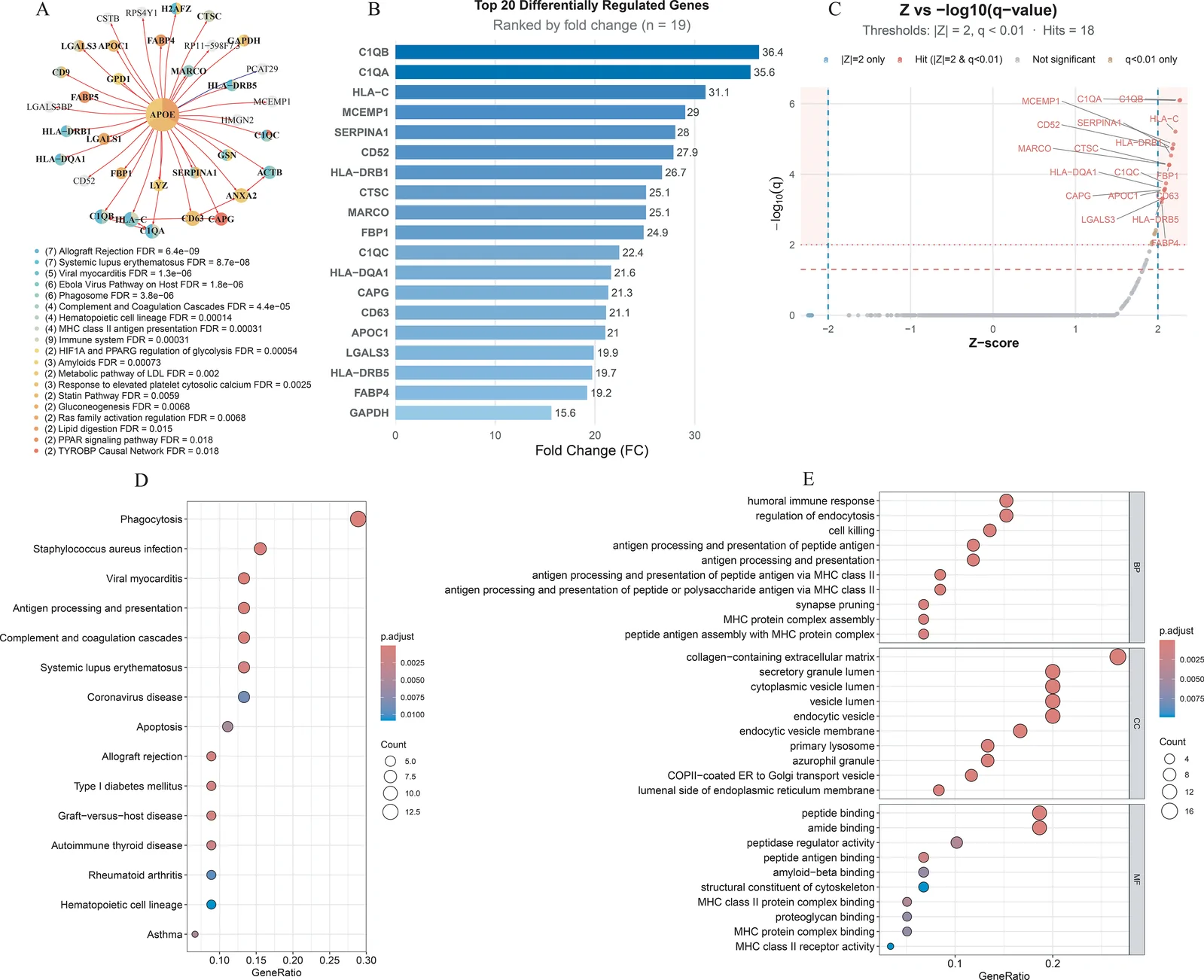
Virtual APOE knockout analysis in macrophages. **(A)** APOE-centered single-cell gene regulatory network generated using scTenifoldKnk analysis. Nodes are colored according to functional pathway classification. **(B, C)** Differentially regulated genes identified following virtual APOE knockout. **(D, E)** KEGG and GO enrichment analyses of the differentially regulated genes following virtual APOE knockout.

Transitioning from observational transcriptomics to predictive mechanistic modeling, we utilized the scTenifoldKnk framework to simulate the biological fallout of an APOE checkpointcollapse. In silico ablation of the APOE node within the macrophage single-cell gene regulatory network (scGRN) triggered sweeping transcriptomic perturbations (**Figure 6A**). The most profoundly altered signatures featured a massive upregulation of the classical complement cascade (including C1QA, C1QB, and C1QC; fold change > 20) and major histocompatibility complex class II elements (HLA-C, HLA-DRB1, HLA-DQA1) (**Figures 6B, C**). Additionally, the targeted deletion induced classical scavenger and pro-fibrotic markers, notably MARCO and LGALS3. Functional enrichment of this perturbed network tightly clustered around phagosome hyperactivation, complement/coagulation cascades, and exaggerated humoral immune responses (**Figures 6D, E**). These simulated outcomes strongly suggest that the physical loss of APOE removes a critical biological brake, precipitating an aggressive, immune-active macrophage phenotype.

### 3.7 *In vitro* validation confirms the essential role of APOE in limiting fibroblast activation

**Figure 7:**
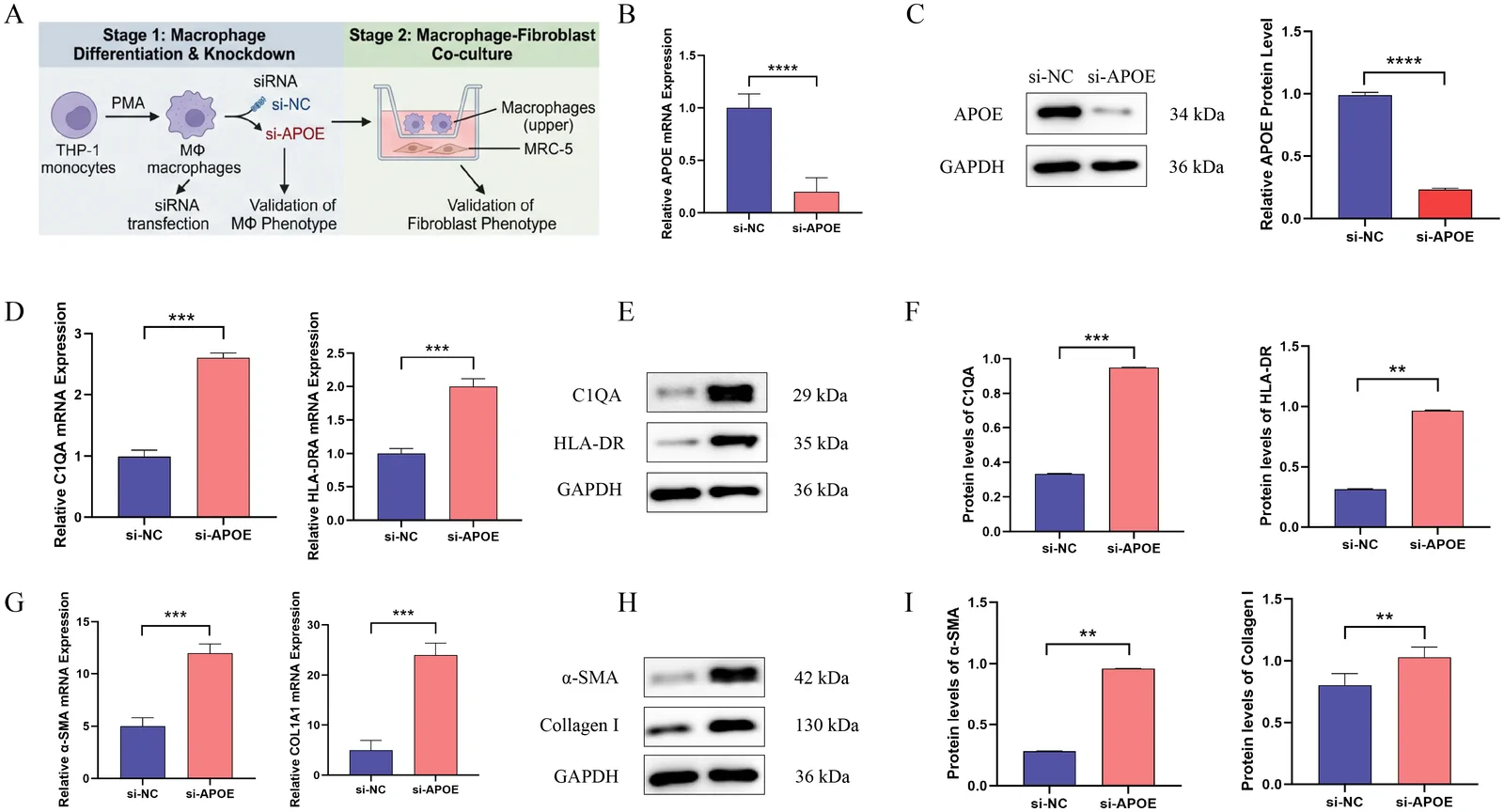
APOE deficiency promotes pathogenic macrophage polarization and fibroblast activation. **(A)** chematic illustration of the macrophage–fibroblast Transwell co-culture model. **(B, C)** Validation of APOE knockdown efficiency in THP-1-derived macrophages at the mRNA **(B)** and protein **(C)** levels 48 h after transfection. **(D–F)** qPCR and Western blot analyses showing increased expression of inflammatory markers C1QA and HLA-DRA following APOE silencing. **(G–I)** qPCR and Western blot analyses demonstrating increased expression of myofibroblast markers ACTA2 and COL1A1 in MRC-5 fibroblasts co-cultured with APOE-deficient macrophages. GAPDH was used as the loading control. Data are shown as mean ± SD (** P < 0.01, *** P < 0.001, **** P < 0.0001).

To empirically validate our computational predictions, we engineered a Transwell co-culture system pairing THP-1-derived macrophages with MRC-5 lung fibroblasts (**Figure 7A**). Targeted siRNA transfection successfully depleted APOE in the THP-1 compartment, achieving robust knockdown at both the transcript and protein levels relative to scrambled controls (**Figures 7B, C**). Exactly as predicted by our virtual knockout model, this physical APOE silencing autonomously triggered a marked escalation in C1QA and HLA-DR expression within the macrophages (**Figures 7D–F**).

Crucially, this exacerbated inflammatory state translated directly into fibrogenic remodeling in the adjacent cellular compartment. MRC-5 fibroblasts co-cultured with APOE-deficient macrophages exhibited a dramatic induction of myofibroblast characteristics, marked by surging α-SMA and collagen I expression across both mRNA and protein assays (**Figures 7G–I**). Together, these *in vitro* data physically corroborate the multi-omics model, confirming that APOE impairment not only derails intrinsic macrophage homeostasis but actively amplifies matrix-depositing fibroblast activation via synergistic paracrine signaling.

To further characterize the phenotypic changes in macrophages prior to fibroblast co-culture, we assessed representative polarization-related markers. Consistent with the bioinformatic predictions, APOE knockdown significantly increased the expression of the pro-inflammatory cytokine TNF-α (~3.2-fold, P < 0.001) and the profibrotic cytokine TGF-β1 (~2.1-fold, P < 0.001) compared with the control group (**Supplementary Figure 8**). These findings suggest that APOE deficiency promotes a pro-inflammatory and profibrotic macrophage phenotype, which may contribute to the enhanced fibroblast activation observed in the co-culture system.

## 4 Discussion

The clinical trajectory of IPF is defined by relentless alveolar epithelial injury and the irreversible destruction of pulmonary architecture through runaway extracellular matrix (ECM) deposition (13, 14). While the current generation of antifibrotic therapeutics-namely pirfenidone and nintedanib-modestly decelerates functional decline, they fundamentally fail to halt the underlying immune-stromal dysregulation or reverse established fibrotic lesions. Bridging the gap between genetic epidemiology and single-cell mechanistics, our study positions APOE as a central metabolic checkpointgoverning macrophage-driven fibrogenesis. Crucially, utilizing a MR framework, we established a robust, protective genetic association from AD traits to IPF risk. This unexpected genetic intersection uncovers a novel translational paradigm: systemic neurodegenerative-associated metabolic programs strongly influence pulmonary fibrotic susceptibility, shifting the clinical focus from strictly organ-specific pathologies to systemic homeostatic networks.

Beyond their distinct anatomical manifestations, AD and IPF share profound aging-related biological vulnerabilities. Both devastating conditions are underpinned by cellular senescence, escalating oxidative stress, mitochondrial failure, and severely impaired proteostasis (15, 16). Systemic metabolic reprogramming is increasingly recognized as a trans-tissue dictator of immune behavior, capable of chronically altering macrophage immunometabolism and inflammatory signaling thresholds across distinct microenvironments (17, 18). Against this backdrop, our initial network screening identified APOE as a prominent molecular candidate bridging these two disease contexts. While APOE emerged as a network hub in line with its well-established role in lipid metabolism, subsequent single-cell analyses and *in vitro* experiments supported its potential involvement in macrophage dysfunction and macrophage-fibroblast crosstalk in IPF. Classically relegated to central nervous system lipid transport and amyloid-beta clearance in AD, APOE is now rapidly emerging as a master regulator of peripheral lipid handling and inflammatory homeostasis (19, 20). Validating this paradigm shift, our pathway enrichment profiles heavily localized to cholesterol efflux, chylomicron remnant clearance, and lipid transport, cementing APOE**’**s role as an indispensable metabolic buffer within the pulmonary nichen.

Notably, this metabolic trajectory within the fibrotic lung draws striking conceptual parallels to the immune landscape of the central nervous system. In the context of AD, APOE is universally recognized as the master transcriptional regulator driving the transition of homeostatic microglia into Disease-Associated Microglia (DAM) (21). Recent single-cell atlases and metabolic studies consistently demonstrate that the neural DAM phenotype heavily relies on APOE induction to buffer extreme amyloid-induced lipid burdens and clear myelin debris (22, 23). Our lung-focused single-cell data highlight a converging paradigm: pulmonary macrophages face a similar metabolic bottleneck when confronting the severe lipid toxicity and oxidative stress inherent to the fibrotic niche. Thus, the APOE metabolic checkpointacts as a universal immune-metabolic sentinel across distinct tissue microenvironments, orchestrating localized cellular fates-whether neurodegeneration or fibrogenesis-in response to overwhelming lipid stress (24).

Spatial and transcriptomic mapping predominantly localized this APOE activity to the macrophage populations residing within the IPF microenvironment. Surprisingly, initial global quantitative analysis revealed that overall APOE transcript abundance did not differ significantly between fibrotic and homeostatic donor macrophages. However, high-resolution analysis at the macrophage-state level revealed that APOE expression was not uniformly distributed across all populations. Instead, APOE expression intensity was relatively increased in the terminal pro-fibrotic macrophage subset, suggesting a potential compensatory transcriptional response under persistent fibrotic stress. Yet, deeper molecular profiling exposed a stark functional paradox. Macrophages sequestered within fibrotic lungs exhibited marked impairment of oxidative phosphorylation, mitophagy, and glutathione metabolism, indicating profound mitochondrial and metabolic dysfunction. Under homeostatic conditions, APOE continuously drives cholesterol efflux and suppresses inflammatory cascades via LXR/RXR-associated signaling circuits (25). However, the unrelenting oxidative stress, continuous epithelial damage, and reactive oxygen species (ROS) bombardment defining the IPF lung likely uncouple this delicate metabolic machinery (26). The mechanisms underlying this functional uncoupling despite preserved APOE transcription are likely multifactorial. First, chronic oxidative stress may induce post-translational modifications of APOE, including protein oxidation, potentially affecting APOE stability, conformation, and lipid-binding capacity (27). Second, alterations in APOE-interacting receptors, such as LRP1 or TREM2, may impair downstream lipid sensing and immune regulatory functions despite preserved APOE expression. The inflammatory and proteolytic milieu of the fibrotic niche may further exacerbate this dysfunction by altering receptor availability and APOE-mediated signaling through mechanisms such as ectodomain shedding (28, 29). Finally, defective mitophagy further exacerbates this crisis by trapping dysfunctional mitochondria within the cell, perpetuating a highly destructive feedback loop of ROS overproduction, lipid peroxidation, and ensuing endoplasmic reticulum stress (30, 31). Thus, despite physically preserved mRNA levels, the APOE metabolic shield exhibits profound transcriptomic dysregulation, suggesting functional impairment. This observation strongly reinforces the concept that IPF metabolic failure stems from system-wide biochemical collapse and intracellular network uncoupling, rather than mere transcriptional loss (32, 33).

Considering the substantial heterogeneity of pulmonary macrophages in IPF, the trajectory inferred by Monocle 3 should be interpreted as a framework describing transcriptomic state organization rather than a definitive differentiation hierarchy. The complementary macrophage subtype analysis demonstrated the coexistence of resident-like, lipid-associated, inflammatory, and fibrosis-associated populations, supporting a model in which multiple macrophage states dynamically contribute to fibrotic remodeling. Consistent with previous studies demonstrating the persistence and pathogenic contribution of monocyte-derived macrophages in pulmonary fibrosis (34), our pseudotime analysis revealed a disease-associated transcriptomic continuum characterized by acquisition of pro-fibrotic features rather than a definitive lineage transition. This trajectory-associated remodeling was characterized by increased expression of SPP1 (osteopontin) and MMP7-both potent and heavily implicated drivers of pathological matrix remodeling (35, 36). While the emergence of SPP1+ macrophages as dominant fibrogenic effectors is increasingly recognized in single-cell atlases, the initiating molecular triggers have remained elusive. We propose that severe intracellular lipid stress and mitochondrial paralysis act as the primary catalysts for this phenotypic shift. Stripped of APOE-mediated lipid regulation, these macrophages succumb to intracellular lipid toxicity, which subsequently ignites powerful inflammatory signaling cascades, including the MAPK and NF-κB pathways. As empirically validated by our *in vitro* data, this metabolic disruption is sufficient to intrinsically trigger a massive release of the canonical M1-associated pro-inflammatory cytokine TNF-α. This metabolic-to-inflammatory crossover effectively locks the cells into a highly pathogenic, mixed pro-inflammatory and pro-fibrotic secretory state, forcing them to continuously churn out TGF-β1, SPP1 and matrix-remodeling mediators that relentlessly fuel the fibrotic niche (37, 38).

High-resolution intercellular communication mapping, supported by validated macrophage and fibroblast marker profiles, revealed how SPP1+ macrophages potentially contribute to tissue remodeling. Among all inferred signaling interactions, macrophage-derived SPP1 emerged as the paramount paracrine signal bombarding local fibroblast populations. Mechanistically, this intensive crosstalk hinges on fibroblastic CD44 receptors and highly specific integrin complexes, notably ITGAV/ITGB1 and ITGAV/ITGB5. Ligation of these surface receptors by SPP1 triggers robust intracellular downstream kinase cascades, including the PI3K/AKT and FAK/SRC pathways, promoting fibroblasts to overcome contact inhibition and undergo phenotypic transformation into synthetic myofibroblasts (39, 40). Concurrently, this integrin engagement serves a secondary physical function: it mechanically amplifies the local release of latent TGF-β tethered within the extracellular matrix, thereby initiating a potent, self-sustaining positive feedback loop of fibrogenesis (41). This precise mechanistic wiring perfectly explains the dense spatial tethering of SPP1+ macrophages to active, proliferating fibroblastic foci frequently observed in clinical IPF histology.

Beyond the SPP1 axis, our supplementary interactome analysis revealed selective remodeling of lipid-handling-associated intercellular communication in IPF, including enhanced fibroblast-to-macrophage PROS1-AXL signaling. The PROS1/TAM receptor axis has been implicated in macrophage efferocytosis, immune regulation, and the clearance of lipid-rich apoptotic cellular debris (42, 43). Although the current CellChat database does not directly model the APOE-LRP1 interaction, the altered PROS1-AXL communication provides additional evidence that lipid-processing demands within the fibrotic niche reshape macrophage-stromal metabolic interactions. These findings are consistent with our central hypothesis that disruption of macrophage lipid homeostasis contributes to pathogenic cellular crosstalk during IPF progression.

To empirically validate this metabolic-inflammatory axis, we bridged in silico computational predictions with targeted *in vitro* cellular assays. Simulating an APOE checkpointcollapse via the scTenifoldKnk virtual knockout framework predicted a massive unleashing of classical complement components-specifically C1QA, C1QB, and C1QC-alongside hyperactivated MHC class II antigen presentation molecules, including the HLA-DR family (44). Physical APOE knockdown in THP-1-derived macrophages faithfully phenocopied this computational forecast. Crucially, even prior to any paracrine interaction with fibroblasts, APOE-deficient macrophages intrinsically shifted toward a hyperactive secretory phenotype, characterized by robust upregulation of both TNF-α and TGF-β1, alongside elevated complement (C1QA) and antigen-presentation (HLA-DR) modules. Consequently, when MRC-5 lung fibroblasts were exposed to these APOE-deficient macrophages in a Transwell co-culture microenvironment, they underwent rapid and aggressive myofibroblast differentiation, characterized by surging α-SMA and type I collagen production. Beyond the SPP1 axis, this hyperactive complement secretion likely acts in synergy to exacerbate stromal remodeling, as C1q-mediated signaling has been actively implicated in directly driving fibroblast activation across multiple chronic fibrotic disorders (45, 46). Consequently, APOE operates as a critical immune-metabolic brake; its failure releases a synergistic inflammatory, complement-driven, and profibrotic storm.

Several limitations of the present study should be acknowledged. Because our MR-based genetic inference was derived primarily from European-ancestry GWAS cohorts, the generalizability of the AD-IPF genetic association across diverse populations requires further cross-ethnic validation. In addition, although the Transwell co-culture system enabled the assessment of soluble macrophage-fibroblast communication, this reductionist model does not fully recapitulate the complex biomechanical properties, extracellular matrix remodeling, and multicellular interactions present in the aged fibrotic lung microenvironment. Although our findings support a role for APOE deficiency in promoting macrophage-driven fibroblast activation, the downstream requirement of the SPP1-CD44 signaling axis remains to be directly validated. Future studies using targeted inhibition approaches, such as SPP1 neutralization or CD44 blockade in macrophage-fibroblast co-culture systems, will be important to establish the causal contribution of this pathway. Furthermore, conclusions regarding macrophage metabolic dysregulation are primarily based on transcriptomic analyses. Direct functional assessments, including cholesterol efflux assays, lipidomic profiling, and mitochondrial metabolic measurements, will be required to further validate the proposed APOE-associated metabolic mechanism. Although Monocle 3 provided insights into macrophage transcriptomic state organization, the absence of raw sequencing data containing spliced and unspliced transcripts prevented RNA velocity analysis. Therefore, the inferred pseudotime trajectories should be interpreted as transcriptomic state continua rather than definitive lineage trajectories. Future studies incorporating appropriate RNA velocity datasets and *in vivo* models, including myeloid-specific Apoe conditional knockout mice subjected to fibrotic challenge or aging conditions, will be necessary to define the spatiotemporal dynamics and therapeutic relevance of APOE-mediated macrophage remodeling. Overall, our findings provide a mechanistic framework linking APOE-associated lipid dysregulation with macrophage-fibroblast communication in IPF; however, therapeutic implications remain hypothetical and require validation in rigorous preclinical models.

## 5 Conclusions

**Figure 8:**
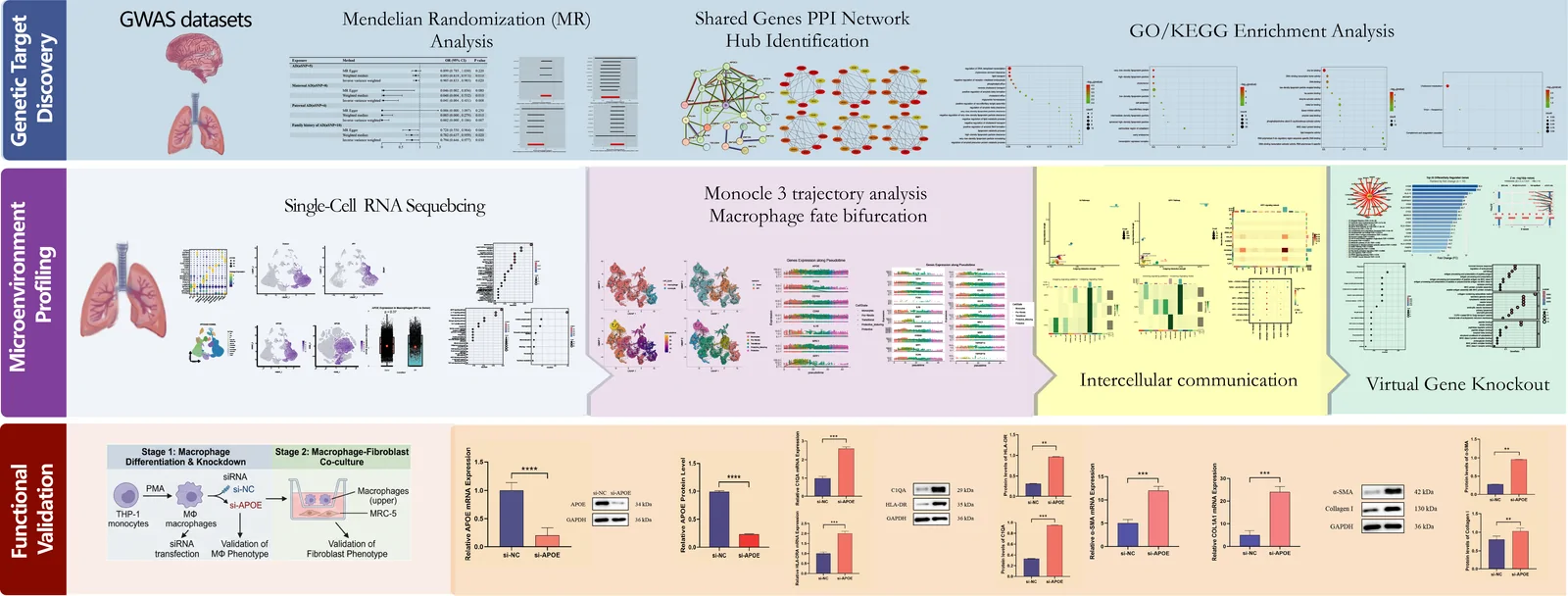
Graphical abstract. Schematic overview of the proposed molecular framework linking APOE-associated macrophage metabolic dysregulation, SPP1-mediated macrophage-fibroblast communication, and pulmonary fibrosis.

In summary, this study delineates the APOE metabolic checkpoint as a fundamental nexus translating Alzheimer’s-associated genetic traits into pulmonary fibrotic risk. We reveal that while baseline transcripts remain stable, the IPF microenvironment is associated with severe transcriptomic dysregulation of APOE-mediated lipid and mitochondrial homeostasis. This putative metabolic impairment licenses macrophages to adopt an aggressive SPP1+ effector phenotype, which subsequently bombards lung fibroblasts via targeted integrin signaling and hyperactive complement cascades. By elucidating this pathogenic transition from metabolic failure to profibrotic inflammation, our findings provide a novel theoretical framework for macrophage biology in fibrotic microenvironments. Nevertheless, establishing the actual therapeutic relevance of targeting this lipid metabolic axis strictly necessitates comprehensive future *in vivo* validation. The proposed mechanistic framework is summarized in **Figure 8**.

## Data Availability

The original contributions presented in the study are included in the article/**Supplementary Material**. Further inquiries can be directed to the corresponding author.

## Supplementary material

Supplementary Figure 1 APOE is identified as a consensus hub candidate in the STRING protein-association network. **(A)** STRING v12.0 protein-association network constructed from 37 candidate genes. After identifier mapping and removal of isolated nodes, the network contained 25 connected nodes and 92 interactions at a combined interaction score threshold of ≥0.400. Node size represents degree, edge width represents the STRING combined interaction score, and node colors indicate APOE, consensus hub candidates, and other connected genes. **(B–G)** Top 10 ranked genes identified by six cytoHubba algorithms: EcCentricity **(B)**, maximum neighborhood component [MNC; **(C)**], closeness **(D)**, degree **(E)**, maximal clique centrality [MCC; **(F)**], and edge percolated component [EPC; **(G)**]. APOE was consistently ranked among the top candidates across all six cytoHubba algorithms. EPC scores were calculated using 1,000 edge-percolation replicates with an edge-removal probability of 0.5. PVRL2 is displayed using the current STRING preferred name NECTIN2.

Supplementary Figure 2 Functional enrichment analyses of shared AD-IPF genes. Bubble plots displaying the top enriched **(A)** Biological Process, **(B)** Cellular Component, **(C)** Molecular Function, and **(D)** KEGG pathways.

Supplementary Figure 3 APOE expression across macrophage cell states. **(A)** UMAP visualization showing the identified macrophage cell states (left) and the corresponding APOE expression pattern across the monocyte–macrophage cell states (right). **(B)** Bubble plot showing the percentage of APOE-positive cells (bubble size) and the mean normalized APOE expression level (color intensity) across the identified macrophage cell states.

Supplementary Figure 4 Independent donor- and IPF-specific Monocle 3 trajectory analyses reveal macrophage transcriptomic state organization. **(A, B)** IPF-specific Monocle 3 trajectory analysis of the monocyte–macrophage compartment. Cells are visualized according to inferred pseudotime ordering **(A)** and annotated cell identities **(B)**. **(C, D)** Donor-specific Monocle 3 trajectory analysis of the monocyte–macrophage compartment. Cells are visualized according to inferred pseudotime ordering **(C)** and annotated cell identities **(D)**. **(E, F)** Pseudotime-associated expression dynamics of macrophage functional genes in IPF and donor samples. The expression patterns of representative macrophage functional programs were evaluated across the inferred pseudotime ordering.

Supplementary Figure 5 Canonical macrophage subtype heterogeneity in IPF lung macrophages. **(A)** UMAP visualization of macrophage subtype annotation based on the macrophage compartment. Six major macrophage populations were identified, including resident tissue macrophages (RTM), lipid-associated macrophages (LA-M), inflammatory macrophages (Inflam-M), fibrosis-associated macrophages (fibrosis-M), anti-inflammatory macrophages (Anti-Inflam-M), and proliferative macrophages (Prolif-M). **(B)** Quantification of macrophage subtype composition showing the relative abundance of each identified population.

Supplementary Figure 6 Canonical marker gene expression across annotated cell populations. Dot plot showing the scaled average expression and the percentage of cells expressing canonical macrophage and fibroblast marker genes across all annotated cell populations in the scRNA-seq dataset. Dot size indicates the percentage of cells expressing each gene in a given cell cluster, and color intensity represents the scaled average expression level.

Supplementary Figure 7 CellChat-based analysis reveals altered lipid-associated ligand-receptor communication between macrophages and fibroblasts in IPF. **(A)** Patient-level comparison of lipid-associated macrophage–fibroblast communication patterns between IPF patients and healthy donors. Six lipid/metabolism-related signaling pathways (ANGPTL, CHEMERIN, GAS, PROS, RESISTIN, and VISFATIN) were evaluated using CellChat based on individual samples. The box plots represent sample-level inferred communication probabilities within the macrophage/monocyte compartment, with boxes indicating the median and interquartile range. **(B, C)** Differential outgoing **(B)** and incoming **(C)** communication activities of lipid-associated pathways in IPF compared with donor controls. Heatmaps display log2 fold changes of inferred pathway activity across major cell populations, including fibroblasts, monocyte/macrophage subsets, endothelial cells, and AT2 cells. **(D)** Predicted lipid-associated ligand-receptor interactions involved in macrophage-fibroblast communication. Bubble plots show inferred ligand-receptor pairs contributing to fibroblast-to-macrophage/monocyte and macrophage/monocyte-to-fibroblast communication, including PROS1-AXL, GAS6-AXL, GAS6-MERTK, RARRES2-CMKLR1, and NAMPT-associated interactions. Dot size represents inferred communication probability, and color intensity indicates interaction strength.

Supplementary Figure 8 Effects of APOE knockdown on inflammatory and pro-fibrotic gene expression in macrophages. **(A, B)** THP-1-derived macrophages were transfected with si-APOE or si-NC, and mRNA expression was analyzed 48 h after transfection. **(A)** Relative TNF-α mRNA levels following APOE knockdown. **(B)** Relative TGF-β1 mRNA levels following APOE knockdown.
