# Supplementary material for: Multi-omics integration identifies APOE as a metabolic regulator of macrophage-fibroblast crosstalk in idiopathic pulmonary fibrosis
Source: Front Immunol. 2026 Aug 18;17:1904638. doi: 10.3389/fimmu.2026.1904638 (PMC13529525; doi:10.3389/fimmu.2026.1904638)
Supplement: Supplementary file 1 [file SupplementaryFile1.docx]

## **Table S1. Sequences of primers used for quantitative real-time PCR**

| Gene | Forward Primer (5' → 3') | Reverse Primer (5' → 3') |
| --- | --- | --- |
| APOE | CTGGTTCGAGCCAATAGTGGAAGAC | CTGGATATGGATGTTGTTGCAGGAC |
| C1QA | CAACCAGCCGCTGTCATTG | GGTAGCCCTTCTTGAAGGAGG |
| HLA-DRA | GACTAAGCGACTCCCACCC | GGGAACCTTCCCATCACCA |
| α-SMA | GTGTTGCCCCTGAAGAGCAT | GCTGGGACATTGAAAGTCTCA |
| Collagen I | GAGGGCCAAGACGAAGACATC | CAGATCACGTCATCGCACAAC |
| TNF-α | CCTCTCTCTAATCAGCCCTCTG | GAGGACCTGGGAGTAGATGAG |
| TGF-β1 | GGCCAGATCCTGTCCAAGC | GTGGGTTTCCACCATTAGCAC |
| GAPDH | GGAGCGAGATCCCTCCAAAAT | GGCTGTTGTCATACTTCTCATGG |
