# Supplementary material for: Multi-omics integration identifies APOE as a metabolic regulator of macrophage-fibroblast crosstalk in idiopathic pulmonary fibrosis
Source: Front Immunol. 2026 Aug 18;17:1904638. doi: 10.3389/fimmu.2026.1904638 (PMC13529525; doi:10.3389/fimmu.2026.1904638)
Supplement: Supplementary file 2 [file SupplementaryFile2.docx]

## **The detailed information of SNPs for AD, maternal AD, paternal AD, and family history of AD**

## **1. The detailed information of 5 SNPs Association with AD**

| SNP | effect_allele.exposure | other_allele.exposure | se.exposure | beta.exposure | pos.exposure | pval.exposure | F |
| --- | --- | --- | --- | --- | --- | --- | --- |
| rs111371860 | T | A | 0.0471 | 0.3811 | 45345787 | 6.10E-16 | 65.46792 |
| rs11668855 | T | C | 0.0926 | 0.5167 | 46452883 | 2.44E-08 | 31.1349 |
| rs429358 | C | T | 0.038 | 1.3196 | 45411941 | 1.00E-200 | 1205.896 |
| rs1882752 | C | G | 0.029 | 0.2303 | 45461342 | 1.95E-15 | 63.06441 |
| rs10425146 | T | G | 0.0792 | 0.6702 | 44730553 | 2.66E-17 | 71.60625 |

## **2. The detailed information of 8 SNPs Association with Maternal AD**

| SNP | effect_allele.exposure | other_allele.exposure | se.exposure | beta.exposure | pos.exposure | pval.exposure | F |
| --- | --- | --- | --- | --- | --- | --- | --- |
| rs6733839 | T | C | 0.000635903 | 0.00589016 | 127892810 | 2.00E-20 | 85.79666 |
| rs10939105 | G | A | 0.00068919 | -0.00385511 | 11023682 | 2.20E-08 | 31.28914 |
| rs1532276 | C | T | 0.000620949 | 0.00366401 | 27466157 | 3.60E-09 | 34.81764 |
| rs554311 | T | C | 0.000680017 | -0.00386668 | 59852569 | 1.30E-08 | 32.33217 |
| rs2965169 | C | A | 0.000625496 | -0.00590044 | 45251156 | 4.00E-21 | 88.98518 |
| rs429358 | C | T | 0.000844117 | 0.0478482 | 45411941 | 1.00E-200 | 3213.097 |
| rs56394238 | G | A | 0.000616377 | 0.00748573 | 45335676 | 6.10E-34 | 147.4937 |
| rs147901416 | A | C | 0.000857421 | 0.00561938 | 45466792 | 5.60E-11 | 42.95231 |

## **3. The detailed information of 4 SNPs Association with Paternal AD**

| SNP | effect_allele.exposure | other_allele.exposure | se.exposure | beta.exposure | pos.exposure | pval.exposure | F |
| --- | --- | --- | --- | --- | --- | --- | --- |
| rs6733839 | T | C | 0.000499313 | 0.00313752 | 127892810 | 3.30E-10 | 39.48436 |
| rs429358 | C | T | 0.000663291 | 0.0248558 | 45411941 | 1.00E-200 | 1404.252 |
| rs4803748 | T | C | 0.00049505 | -0.00286042 | 45247048 | 7.60E-09 | 33.38561 |
| rs56394238 | G | A | 0.00048412 | 0.00365176 | 45335676 | 4.60E-14 | 56.8979 |

## **4. The detailed information of 10 SNPs Association with Family history of AD**

| SNP | effect_allele.exposure | other_allele.exposure | se.exposure | beta.exposure | pos.exposure | pval.exposure | F |
| --- | --- | --- | --- | --- | --- | --- | --- |
| rs147901416 | A | C | 0.0099 | 0.0547 | 45466792 | 3.60E-08 | 30.52822 |
| rs2965169 | C | A | 0.0101 | -0.1021 | 45251156 | 6.34E-24 | 102.1894 |
| rs3845261 | T | C | 0.0099 | 0.0545 | 5008236 | 4.01E-08 | 30.30539 |
| rs3851179 | C | T | 0.01 | 0.0706 | 85868640 | 1.76E-12 | 49.84328 |
| rs429358 | C | T | 0.0091 | 0.5686 | 45411941 | 1.00E-200 | 3904.165 |
| rs56394238 | G | A | 0.0099 | 0.1315 | 45335676 | 5.26E-40 | 176.4324 |
| rs6605556 | G | A | 0.0099 | -0.0543 | 32583099 | 3.78E-08 | 30.08337 |
| rs6733839 | T | C | 0.0099 | 0.108 | 127892810 | 1.06E-27 | 119.0075 |
| rs679515 | C | T | 0.0098 | -0.0572 | 207750568 | 5.29E-09 | 34.06726 |
| rs7384878 | T | C | 0.01 | 0.0644 | 99932049 | 1.29E-10 | 41.47334 |
